# Supplementary material for: An optoacoustic field-programmable perceptron for recurrent neural networks
Source: Nat Commun. 2024 Apr 16;15:3020. doi: 10.1038/s41467-024-47053-6 (PMC11021513; doi:10.1038/s41467-024-47053-6)
Supplement: Supplementary file 1 — Supplementary Information [file 41467_2024_47053_MOESM1_ESM.pdf]

Supplementary information: An optoacoustic field-programmable perceptron for recurrent neural networks

Steven Becker[1,2], Dirk Englund[3], and Birgit Stiller[1,2,*]

[1]Max-Planck-Institute for the Science of Light,
Staudtstr. 2, 91058 Erlangen, Germany
[2]Department of Physics, Friedrich-Alexander-Universität Erlangen-Nürnberg
Staudtstr. 7, 91058 Erlangen, Germany
[3]Research Laboratory of Electronics, Massachusetts Institute of Technology,
Cambridge, Massachusetts 02139, USA

[*] Corresponding author. Email: birgit.stiller@mpl.mpg.de

Table of Contents

S1 Experimental setup 3

S2 Temporal calibration 3

S3 Intrinsic correlation check 5

S4 Performance evaluation 7

S5 Scalability of OREO-based optical RNN 9

S6 All-optical control 11

S7 OREO with a highly nonlinear fiber 13

S8 Theory of OREO 15

S9 Numerical study of the *abc*-pattern prediction 19

S10Comparing: OREO vs RNN 21

S1 Experimental setup

This section presents the experimental setup described in the Method section of the main text.

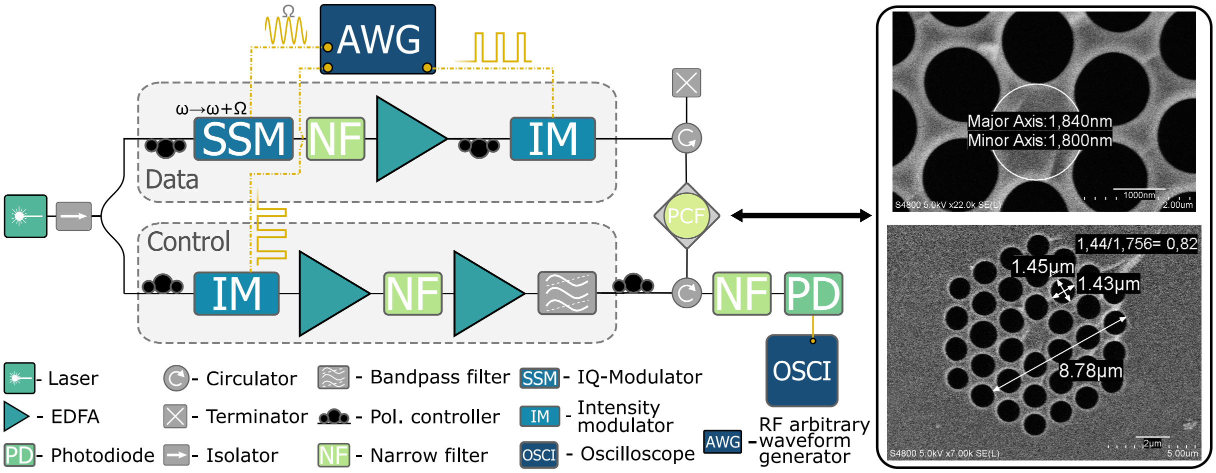

Supplementary Fig. 1: Illustration of the setup used to demonstrate the recurrent optoacoustic operator. The bandwidth of the oscilloscope and photodiode are 16 GHz and 12 GHz, respectively. We introduce the Brillouin process inside photonic crystal fiber (PCF), which could be replaced with any other waveguide or an on-chip device.

S2 Temporal calibration

The temporal calibration ensures that only one data-control pulse pair interacts in the optical fiber. The isolation of a single stimulated Brillouin scattering (SBS) process allows us to rule out any parasitic interactions, e.g., $a_{\mathrm{D},1}$ interacts with $a_{\mathrm{C},2}$. In addition, the acoustic wave b_1 exists only at a single location in the optical fiber. Note that for future applications of OREO, it might not be necessary to isolate the interactions that strictly.

The selected PCF has a length of 40 cm, corresponding to a pulse travel time of about 2 ns. Hence, by separating two pulses with a deadtime of 2.5 ns, one ensures single pulse interactions. Furthermore, the path lengths of the data and control branches differ in our

setup due to the different devices in the corresponding path. Consequently, we compensate for the different path lengths by electrically delaying the control pulses with the arbitrary waveform generator (AWG) (see Supplementary Figure 4 of the main text).

We sweep the temporal offset of the control pulses, while keeping the data offset constant. In order to extract the optimal control offset, we measure the depletion of the data pulses $a_{\mathrm{D},1}$, $a_{\mathrm{D},2}$, and $a_{\mathrm{D},3}$ for the case, where only one of the control pulses $a_{\mathrm{C,i}}$ is active. This allows us to isolate the interaction between the different pairs. The blue solid curve in the Supplementary Figure 2a represents the desired interaction $a_{\mathrm{D},1} \leftrightarrow a_{\mathrm{C},1}$. The dashed grey lines in the same plot represent the interaction of $a_{\mathrm{C},1}$ with either $a_{\mathrm{D},2}$ or with $a_{\mathrm{D},3}$. As discussed, these interactions are not of interest for the demonstration of OREO. Supplementary Figure 2b and c show the results, where $a_{\mathrm{C},2}$ and $a_{\mathrm{C},3}$ only are active, respectively. The colored curve in both Supplementary Figures shows the behavior of the corresponding data pulses $a_{\mathrm{D},2}$ and $a_{\mathrm{D},3}$. A green square in each plot marks the region, where we observe the desired interaction $a_{\mathrm{D,i}} \leftrightarrow a_{\mathrm{C,i}}$ and no parasitic interaction. This region covers a time span of about 2 ns translating to the length of the fiber. Consequently outside this zone, the data pulse $a_{\mathrm{D,i}}$ or the control pulse $a_{\mathrm{C,i}}$ takes only partially part in the SBS-process reducing its efficiency. Based on the results presented in Supplementary Figure 2, we chose 149 ns as the offset for the control pulses.

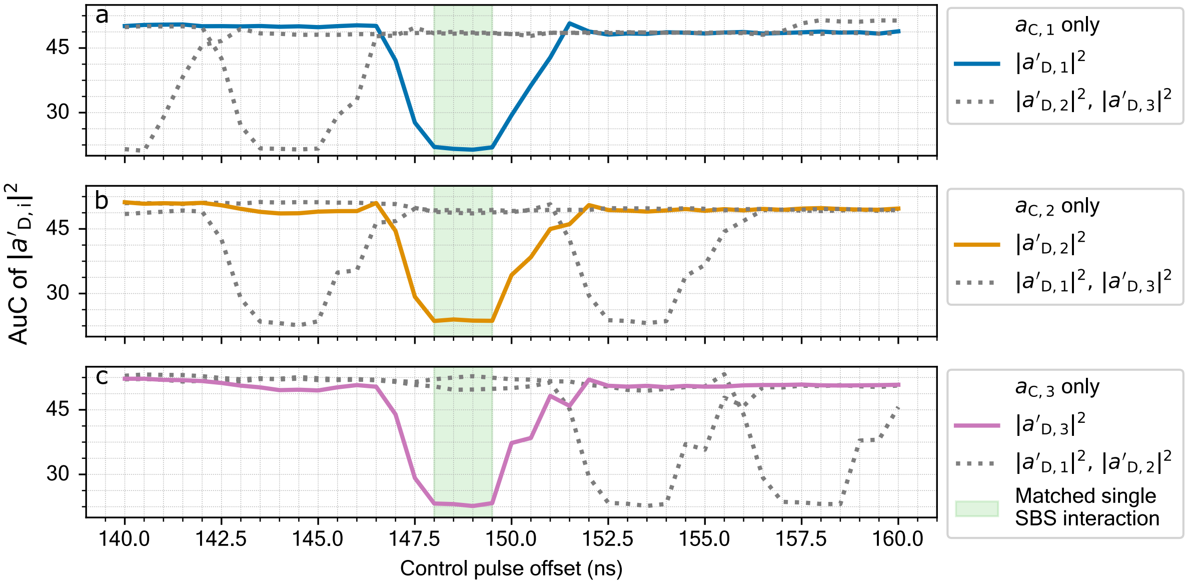

Supplementary Fig. 2: Results of OREO's temporal calibration to ensure that only a single and a matched data-control pulse pair interacts in the optical fiber. a, b, and c study the interaction of $a_{\mathrm{C},1}$, $a_{\mathrm{C},2}$ and $a_{\mathrm{C},3}$ with the different data pulses, respectively. In each plot, the colored solid line marks the desired interaction, e.g., in A it is $a_{\mathrm{D},1} \leftrightarrow a_{\mathrm{C},1}$. Moreover, the gray dashed lines represent unwanted interactions, e.g. in a these are $a_{\mathrm{D},2} \leftrightarrow a_{\mathrm{C},1}$ and $a_{\mathrm{D},3} \leftrightarrow a_{\mathrm{C},1}$. Possible offset values for the control pulses, where OREO performs only the desired isolated and matched interactions $a_{\mathrm{D,i}} \leftrightarrow a_{\mathrm{C,i}}$ are covered by the green rectangular in each plot.

S3 Intrinsic correlation check

The all-optical control is one of OREO's unique features. In order to validate that the connection between the different data pulses is induced by the SBS-process alone, we check the correlation of the different data pulses. Therefore, we sweep the amplitude of data pulses $a_{\mathrm{D},1}$ and $a_{\mathrm{D},2}$, while checking the impact on all subsequent data pulses. We perform this study twice with different settings. In the first sweep, the control pulses are turned off and in a second sweep, the control pulses are on but temporally detunded by 351 ns to the optimal offset.

If we sweep the amplitude of the data pulse $a_{\mathrm{D},1}$, we can observe a small impact on the

output pulses $a'_{\mathrm{D},2}$ and $a'_{\mathrm{D},3}$ as shown in Supplementary Figure 3a and c, respectively. The increase of the output amplitude $a'_{\mathrm{D},2}$ for lower values of $a_{\mathrm{D},1}$ (see Fig. 3a) could be caused by the RF-amplifier which is placed between the arbitrary waveform generator (AWG) and the intensity modulator, which is used for the pulse generation. If the electrical amplitude of the data pulse $a_{\mathrm{D},1}$ decreases the RF-amplifier has more energy to feed into the subsequent data pulse $a_{\mathrm{D},2}$. This could also explain, why the amplitude of $a'_{\mathrm{D},3}$ decreases at the same time (see Fig. 3c). The same effect can most probably be observed for the sweep data pulse $a_{\mathrm{D},2}$. Here, the output amplitude of the subsequent data pulse $a'_{\mathrm{D},3}$ also increases for lower input values of $a_{\mathrm{D},2}$ (see Fig. 3d). In addition, we do not observe any effect of $a_{\mathrm{D},2}$ on the output $a'_{\mathrm{D},1}$ as illustrated in Supplementary Figure 3b, indeed, the data pulse remains almost constant. An overall drift of the system could explain, why the AuC value is slightly below 1. Moreover, if one compares the two measurements in Supplementary Figure 3b, one notices that the detuned control signal slightly depletes the data signal. Most probably this is could be an effect of the EDFA's amplified spontaneous emission (ASE).

In comparison to the results discussed in the main text, we can conclude that the measured intrinsic correlation are neglectable. However, one should keep in mind the data pulse depletion induced by the ASE of the EDFA, which might becomes stronger for higher pump powers of the EDFA.

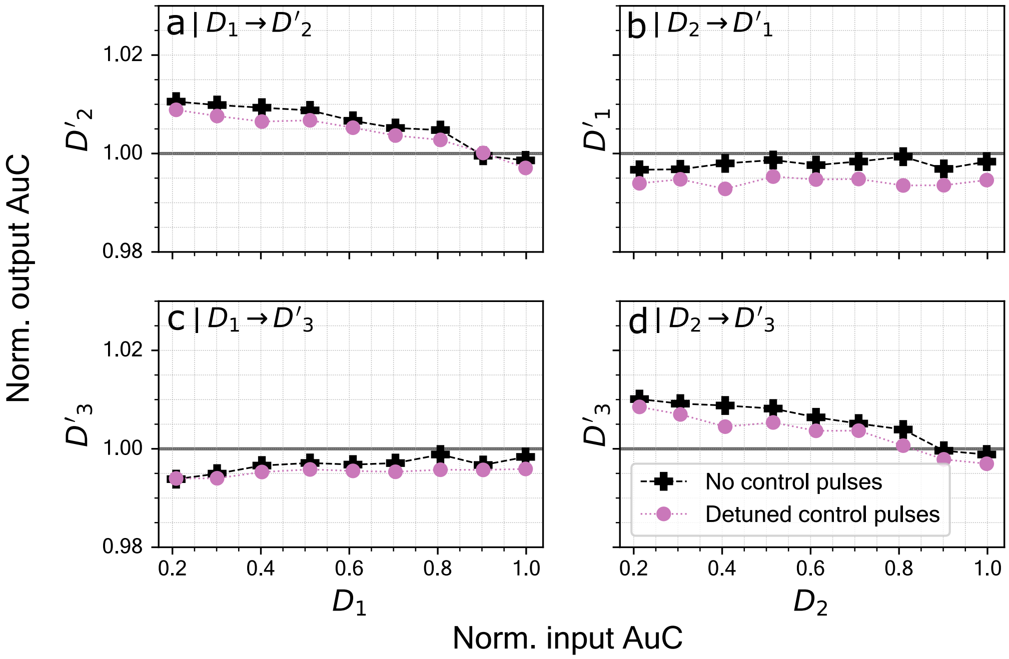

Supplementary Fig. 3: Results of the intrinsic correlation check of OREO. In a and c we can see a small correlation between the input amplitude of the data pulse $a_{\mathrm{D},1}$ and the output amplitudes $a'_{\mathrm{D},2}$ and $a'_{\mathrm{D},3}$, respectively. The reason for this dynamic could be the RF-amplifier located between the AWG and the intensity modulator. A similar dynamic between subsequent pulses occurs if we change the data pulse $a_{\mathrm{D},2}$ and check its impact on the output $a'_{\mathrm{D},3}$ (see d). Again the output amplitude increases for lower inputs. As expected, we can see no connection between the input of $a_{\mathrm{D},2}$ and the output $a'_{\mathrm{D},1}$. However, we see that the EDFA, although detuned, reduces for all four cases the amplitude of the output data pulses. This effect might result from the EDFA's amplified spontaneous emission (ASE). Overall, the intrinsic correlation is significantly lower than the one induced via OREO and can, therefore, be neglected.

S4 Performance evaluation

This section asses the performance of OREO in terms of its speed and energy consumption. The key parameters of OREOs performance are the deadtime dt between two pulse pairs, and the pulse width τ.The lower boundary of the deadtime can be estimated as $dt > \frac{L}{n_{\mathrm{eff}} c_0}$, where L is the waveguide's length, n_{eff} is it effective refractive index, and c_0 is the speed of light. In total, the overall number of recurrent operations (ROPS) which can be achieved

in one second is:

$$C_{\mathrm{SF}} = \frac{1}{dt + \tau}. \tag{1}$$

For a short fiber, one could operate OREO with a deadtime of $dt = 500\,\mathrm{ps}$ and a pulse width of $\tau = 250\,\mathrm{ps}$, yielding $C_{\mathrm{SF}} = 1.33\,\mathrm{GROPS\,s^{-1}}$. The frequency selectiveness of Brillouin scattering allows OREO to process M frequencies simultaneously, extending the performance of OREO further to $C_{\mathrm{MF}} = M \cdot C_{\mathrm{SF}}$. The number of frequencies that can be utilized by the scheme depends on the spectral width of the pulses. For example, a channel separation of 3.6 GHz and a photodetector with a bandwidth of 100 GHz boost the performance of the discussed example by a factor of 28 to $C_{\mathrm{MF}} \approx 28 \cdot C_{\mathrm{SF}} = 36.4\,\mathrm{GROPS\,s^{-1}}$.

The current approach of OREO requires about $P_{\mathrm{pulse}} = 126\,\mathrm{mW}$ of optical power per control pulse $a_{\mathrm{C,i}}$ in the PCF, translating to a pulse energy of $E_{\mathrm{pulse}} = 126\,\mathrm{nJ}$ for the applied repetition rate of 1 MHz. Hence, OREO has an energy efficiency of about $\eta \approx 8\,\mathrm{MROPS\,J^{-1}}$.

A control pulse recycling scheme can enhance OREO's energy efficiency significantly. Such a scheme can be implemented because a control pulse is not depleted through the SBS process, but amplified. Hence, it offers the possibility to re-use it for subsequent computational steps. In addition, the experimental scheme presented offers also an output for the control pulse. In the use case discussed above, a 10 cm delay line between the control input and output of OREO would already be enough for implementing a recycling scheme. Under the assumption of an low loss recycling scheme, where the loss is compensated through the control pulse amplification of the SBS process, the control pulse recycling could increase the efficiency by nine orders of magnitude:

$$\begin{aligned} &\eta_{\mathrm{recy}} = \frac{1}{(dt + \tau)\, E_{\mathrm{pulse}}} = 2.3\,\frac{\mathrm{PROPS}}{\mathrm{J}} \approx 10^9 \cdot \eta, \\ &\text{using} \quad dt = 2.5\,\mathrm{ns},\ \tau = 1\,\mathrm{ns} \end{aligned} \tag{2}$$

For OREO with a long short-term memory that employs a deadtime of $dt = 500\,\text{ps}$ and a pulse width of $\tau = 250\,\text{ps}$ the computational efficiency can be increased to $\eta_{\text{recy}} = 10.6\,\text{PROPS}\,\text{J}^{-1}$. Note that a waveguide with a high optoacoustic gain could further increase the efficiency of OREO.

S5 Scalability of OREO-based optical RNN

In the following we give an estimate on the scalability of an optical RNN using OREO. Therefore, we have to distinguish between different types of architectures of the underlying optical neural network (ONN).

Feed-forward (FF) architecture This type of ONN feeds the output of layer n directly into layer $n + 1$ without amplifying the light between the layers. For instance, the FF-arch. is used by[1].

Supply light (SL) architecture This type of ONN uses the output of layer n to manipulate a supply light channel which is fed into layer $n + 1$. The power in the supply light channel is constant for all layers. For example, the SL-arch. is used by[2].

Optical-Digital-Optical (ODO) architecture This type of ONN converts the output of layer n into the digital domain. The digital information is then transferred back to the optical domain for layer $n + 1$, for instance, via Mach-Zehnder modulators or optical attenuators. Such an approach is used by[3].

In addition, we have to distinguish if the signal is detected with a direct or a homo-/heterodyne detection scheme. In the latter case, the power of the local oscillator P_{LO} must also be taken into account. The minimum input power $P_{\min}$ that an detector can detect depends on its Noise-Equivalent Power and its bandwidth. The maximum number

of RNN layers N for a given input power P_{in} of an OREO-based RNN, where each layer has an insertion loss α_{l}, is given by Table S1.

Table S1: Estimation of the maximum number of RNN layers N, considering the the insertion loss of a layer α_{l}, the input power P_{in}, the minimum detectable power of the detector P_{min}, and the local oscillator power P_{LO}. We assume the minimum signal power detectable with homo-/heterodyne detection to be $P_{\text{min}}^{\text{HD}}(P_{\text{LO}}) \approx (P_{\text{min}})^2/(2P_{\text{LO}})$ (compare[4]). The SL-arch. and the ODO-arch. are independent (Ind.) of the input power P_{min}.

Detection	FF-arch.	SL-arch. and ODO-arch.
Direct	$N = (P_{\text{in}} - P_{\text{min}})/\alpha_{\text{l}}$	Ind. of P_{in} if: $P_{\text{min}} < (P_{\text{in}} - \alpha_{\text{l}})$
Homo-/ Heterodyne	$N = \left(P_{\text{in}} - P_{\text{min}}^{\text{HD}}(P_{\text{LO}})\right)/\alpha_{\text{l}}$	Ind. of P_{in} if: $P_{\text{min}}^{\text{HD}}(P_{\text{LO}}) < (P_{\text{in}} - \alpha_{\text{l}})$

The current design of OREO has an intrinsic insertion loss of 2 dB due to the used circulators. In addition, we use the depletion of a single pulse interaction (SPI) to estimate the optical loss through OREO, yielding $\approx$ 3 dB. Note that this value depends on the chosen configuration of OREO (see Figure 2 in the main text). A complete layer of an RNN is formed if OREO is combined with a matrix multiplication and a nonlinear activation function, which we assume to have a loss of 1.32 dB as reported by[1]. Hence, the total insertion loss per layer is $\alpha_{\text{l}} = 6.32$ dB. With the minimum detection power $P_{\text{min}} \approx -24$ dBm of the used photodetector and the initial input power $P_{\text{in}} \approx 0$ dBm, one could realize four layers. By comparison, the current maximum number of layers reported in an ONN without transferring information back to the digital domain is three[1;2].

In order to increase the number of layers of an OREO-based RNN in the future, one could use a lower SPI-depletion value. For instance, one could convert only 10 % of the data pulse into the acoustic domain, yielding a loss of $\approx$ 0.5 dB. This would yield a

maximum number of ≈ 6 layers that could be realized. Another two layers could be added by replacing the current Newport 12 GHz photodetectors[5] with 10 GHz photodetectors manufactured by Thorlabs[6]. Eventually, one can exchange direct detection with homo-/heterodyne detection which has been already used in[1]. In this case, one could push the maximum number of layers to ≈ 16, considering a local oscillator power of $P_{\mathrm{LO}} = 1\,\mathrm{mW}$.

S6 All-optical control

The control pulses $a_{\mathrm{C,i}}$ can be used to tune the strength of the optoacoustic interaction. Known from an optoacoustic memory[7;8], the power of the control pulses dictates the degree of depletion and, therefore, the relative amount of acoustic that is created. Hence, the control pulses can be used to optimize the interaction for the desired need or to modify the light-sound interaction on a pulse-by-pulse level.

In the following, we study the all-optical control with three data-control pairs (dead-time 2.5 ns), where we vary the amplitude of either the control pulse $a_{\mathrm{C,1}}$ or $a_{\mathrm{C,2}}$, while keeping the remaining control pulse constant (see Fig. 4a). The results for four amplitude scales, namely, full ($\alpha_{Ci} = 1$), three quarters ($\alpha_{Ci} = 0.75$), half ($\alpha_{Ci} = 0.5$), and one quarter ($\alpha_{Ci} = 0.25$) are shown in Supplementary Figure 4.

The amplitude of $a_{\mathrm{C,i}}$ influences the degree of depletion of the data pulse $a_{\mathrm{D,i}}$, and therefore the level of the acoustic wave b_i which we create. Hence, we can change the amplitude of b_i by changing the amplitude of $a_{\mathrm{C,i}}$. In addition, due to the coupling of the optical and acoustic fields, previous generated acoustic waves b_k, $k < i$ are also influenced by $a_{\mathrm{C,i}}$ after the Brillouin process, yielding the dependence $b_k(a_{\mathrm{C,k}}, a_{\mathrm{C,i}})$. Hence, we can use the interaction $a_{\mathrm{D,1}} \leftrightarrow a_{\mathrm{D,1}}$ to isolate the impact of b_1 on subsequent SBS processes if we sweep the amplitude $a_{\mathrm{C,1}}$. The amplitude sweep of the control pulse $a_{\mathrm{C,1}}$ (see Fig. 4b to e) displays the impact of the acoustic interference when one studies the interaction

$a_{\mathrm{D},1} \rightarrow a'_{\mathrm{D},2}$. In the process of reducing the control pulse amplitude, one can see the transition from an inhibition to annihilation to amplification of the interaction $a_{\mathrm{D},2} \leftrightarrow a_{\mathrm{C},2}$ in the plots B, C, and D, respectively. An equivalent dynamic can be observed for the acoustic link $a_{\mathrm{D},1} \rightarrow a'_{\mathrm{D},3}$, which is influenced by the acoustic interference of b_1 and b_2. Here, the acoustic link first amplifies the interaction of $a_{\mathrm{D},3} \leftrightarrow a_{\mathrm{C},3}$, then annihilates it and, finally, inhabits it as one can see in the plots B, D, and E, respectively. Next, the acoustic wave b_1 serves the acoustic link $a_{\mathrm{D},2} \rightarrow a'_{\mathrm{D},3}$ as an amplifier because the degree of depletion of $a_{\mathrm{D},3}$ shrinks for higher control pulse attenuations α_{C1} (see Fig. 4b to e). Next, the amplitude sweep of control pulse $a_{\mathrm{C},2}$ (see Fig. 4f to i) reveals information about the influence of the acoustic waves b_1 and b_2. Although we only change the amplitude of $a_{\mathrm{C},2}$, the coupling of the optical and acoustic domains influences the level of b_1 as well. This interaction inhibits the acoustic link between $a_{\mathrm{D},1} \rightarrow a'_{\mathrm{D},3}$ because the depletion of $a'_{\mathrm{D},3}$ increases with lower amplitudes of $a_{\mathrm{C},2}$ (see Fig. 4f). In addition, the acoustic link $a_{\mathrm{D},2} \rightarrow a'_{\mathrm{D},3}$ also shows the effect of the acoustic interference between b_1 and b_2: for $\alpha_{C1} = 1$, the SBS-process is inhibited; for $\alpha_{C1} = 0.75$ it is annihilated; and for $\alpha_{C1} = 0.5$ it is amplified (see Fig. 4f to h, respectively).

Overall, the control pulses $a_{\mathrm{C,i}}$ serve OREO as an additional degree of freedom to manipulate the recurrent operation on pulse-by-pulse basis.

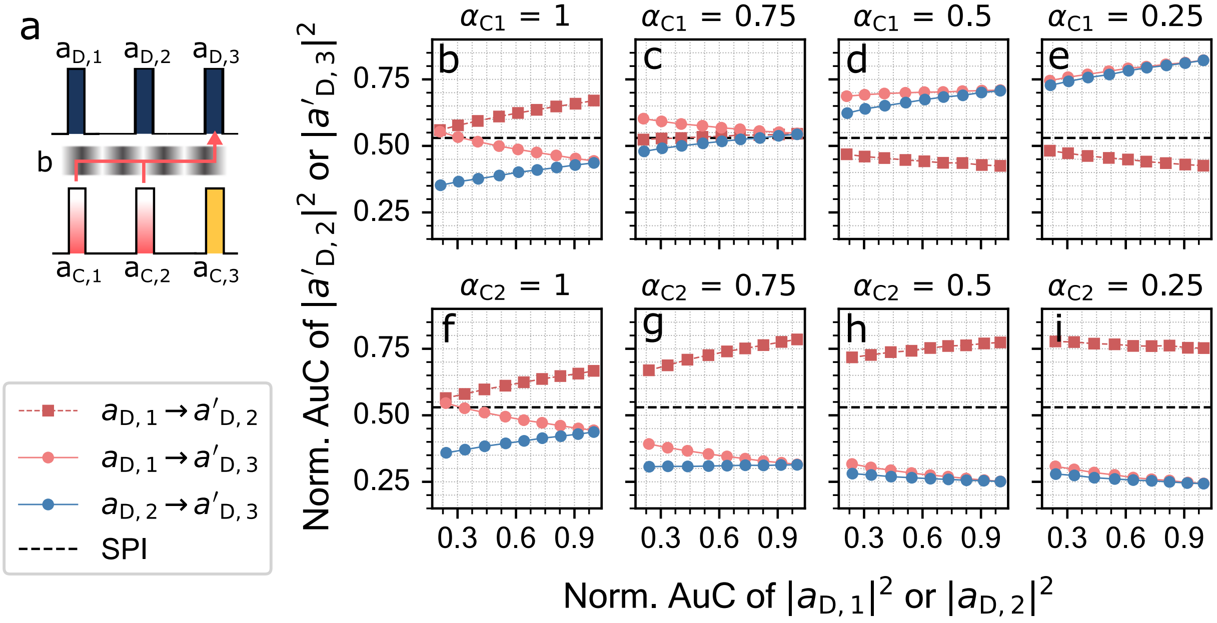

Supplementary Fig. 4: Study of OREO's capability to control the recurrent operation completely optically on pulse-by-pulse basis. The pulse pairs are seperated by a deadtime of $dt = 2.5\,\text{ns}$. a - Schematic illustration of the control pulse sweep that investigates the impact of different control pulse amplitudes on the recurrent interaction. b to e - Experimental results of reducing the amplitude of control pulse $a_{\text{C},1}$. The relative change in control amplitude is given by α_{C1}. This measurement isolates the impact of the acoustic wave b_1 on subsequent SBS processes while b_2 and b_3 remain constant. f to i - Experimental results of reducing the amplitude of the control pulse $a_{\text{C},2}$. This measurement isolates the combined impact of the acoustic waves b_1 and b_2.

S7 OREO with a highly nonlinear fiber

In the following, we demonstrate OREO in a $\approx 43\,\text{cm}$ long highly nonlinear fiber (HNLF). Analogous to the PCF measurement, we launch three consecutive data-control pulse pairs into the fiber and vary the amplitude of $a_{\text{D},1}$ and $a_{\text{D},2}$ (see Supplementary Figure 5a). In contrast to the PCF study, we investigate OREO's dynamic without a frequency detuning, i.e., the frequency difference between data and control pulses matches the Brillouin frequency. Hence, we drop the influence of the acoustic phase. We study the dynamic again for different deadtimes. The recurrent dynamic is less complex than that of the PCF

due to the lack of acoustic interference. As we can see from Supplementary Figure 5b, each SBS process is enhanced by the previous ones. As a result the output amplitude of $a'_{\mathrm{D,2}}$ is higher than that of $a'_{\mathrm{D,3}}$ for all interactions. In contrast to the PCF, this dynamic does not change for different deadtimes (see from Supplementary Figure 5c). The overall higher output amplitudes $a'_{\mathrm{D,i}}$ for the different acoustic links ($a_{\mathrm{D,i}} \rightarrow a'_{\mathrm{D,j}}$) can be explained with the decay of the acoustic wave. Although the link $a_{\mathrm{D,1}} \rightarrow a'_{\mathrm{D,3}}$ touches the acoustic lifetime limit, we are still able to observe a recurrent interaction. This link ($a_{\mathrm{D,1}} \rightarrow a'_{\mathrm{D,3}}$) vanishes clearly as soon as we separate the pulse pairs by the acoustic lifetime (see from Supplementary Figure 5d). In this case, we can only observe nearest neighbour interactions $a_{\mathrm{D,i}} \rightarrow a'_{\mathrm{D,i+1}}$. OREO achieves in our HNLF a maximum dynamic range of $\approx 20\,\%$. A numerical study of the HNLF behavior is depicted in Supplementary Figure 8.

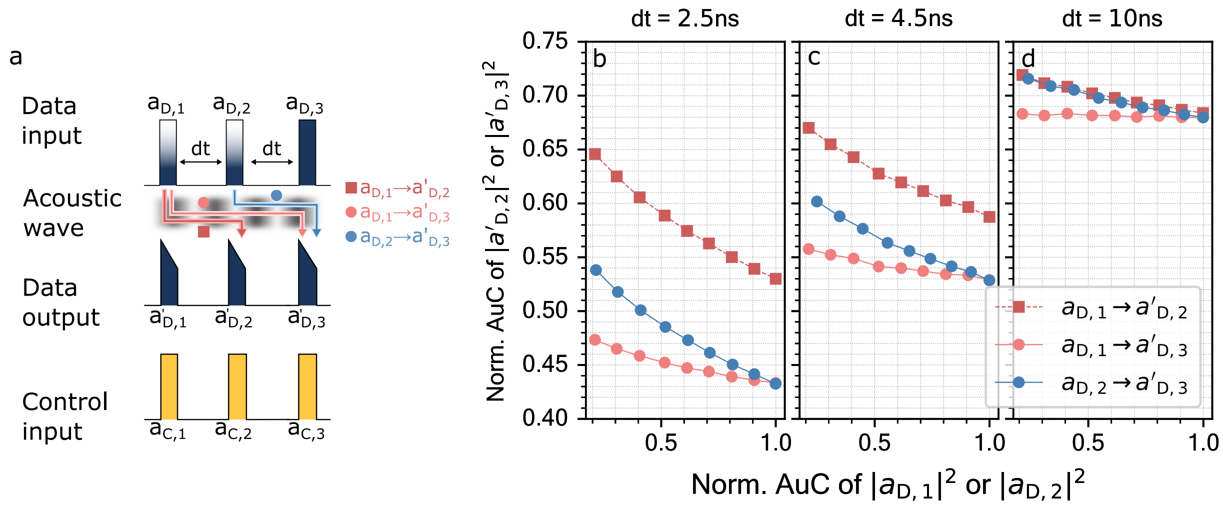

Supplementary Fig. 5: Observing OREO's optoacoustic linking in a HNLF. a - Schematic illustration of the amplitude sweep that investigates how different optical states are passed between the optical data pulses $a_{\mathrm{D,i}}$ via an acoustic wave *b*. b to d - Experimental results of the amplitude sweep in a HNLF. While $a_{\mathrm{D,1}}$ and $a_{\mathrm{D,2}}$ are changed, their impact on the subsequent pulses $a_{\mathrm{D,2}}$ and $a_{\mathrm{D,3}}$ are studied for different deadtimes *dt*. Each interaction $a_{\mathrm{D,i}} \leftrightarrow a_{\mathrm{C,i}}$ creates an acoustic wave that interacts with pre-exisiting ones, eventually, effecting following SBS processes.

S8 Theory of OREO

OREO employs stimulated Brillouin scattering (SBS), which is well-studied in literature[9;10;11;12;13]. In the following, we use these references to elaborate on the theoretical foundations of OREO. Note that we restrict ourselves to backward SBS.

SBS is a third-order nonlinear effect that describes the coherent interaction of light with sound. This interaction is established by two effects, namely, electrostriction and the photoelastic effect. Electrostriction labels the creation of dipole moments inside a medium by a light field. The dipole moments experience an attractive force, pulling them towards the electromagnetic field and changing the medium's density. Eventually, the density changes form an acoustic wave, i.e., density wave, that changes the dielectric properties of the medium due to the photoelastic effect. The optical field experiences the periodic nature of the acoustic wave as a grating, from which it scatters inelastically. The scattered light is red or blue shifted by the Brillouin frequency Ω of the acoustic field and depends on the propagation direction of the acoustic wave. The Brillouin frequency Ω lays in the range of several gigahertz. Furthermore, the initial and the backscattered light fields interfere with each other and, thereby, accelerate the forming of the acoustic wave, which in turn increases the amount of light that is backscattered (see Fig. 6). Hence, this feedback loop forms a stimulated process that significantly enhances the efficiency of Brillouin scattering. Indeed, stimulated Brillouin scattering is several orders of magnitude stronger than the Raman- or the Kerr-effect[9].

According to reference[9], the dynamics of the SBS process can be described by the one-dimensional optical and acoustic wave equations, which are noted in equations (3)

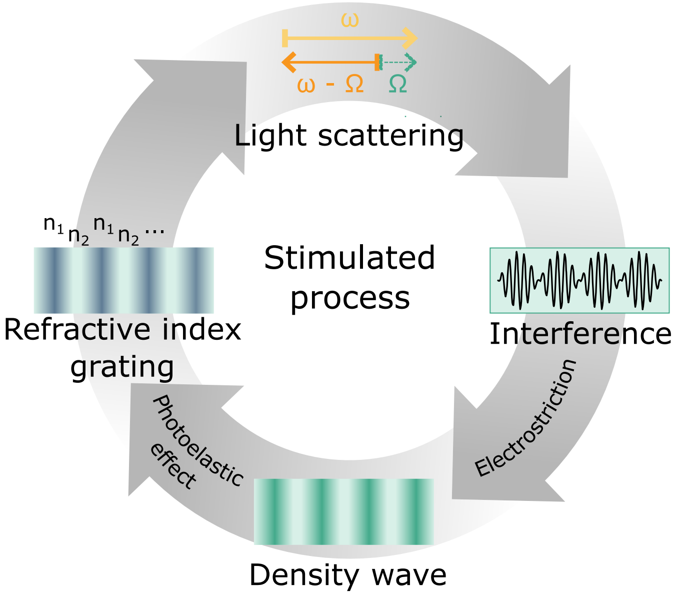

Supplementary Fig. 6: Schematic illustration of the feedback loop between the acoustic and optical waves. An initial light wave scatters from acoustic phonons and is shifted by the Brillouin frequency Ω. The initial and backscattered light fields interfere with each other and create a moving interference pattern. Electrostriction transforms this interference pattern to a moving density wave inside the waveguide, which changes the optical properties of the medium due to the photoelastic effect. The resulting density wave enhances the scattering efficiency and, thus, enables a stimulated process.

and (4), respectively.

$$\frac{\partial^2}{\partial z^2}E - \frac{n^2}{c_0^2}\frac{\partial^2}{\partial t^2}E = \frac{4\pi}{c_0^2}\frac{\partial^2}{\partial t^2}P, \tag{3}$$

$$\frac{\partial^2}{\partial t^2}b - \Gamma'\frac{\partial^2}{\partial z^2}\frac{\partial}{\partial t}b - v_{\mathrm{ac}}\frac{\partial^2}{\partial z^2}b = \nabla\vec{f}. \tag{4}$$

The set of equations (3) and (4) use the one dimensional electric field E, the acoustic field b, the refractive index n, the speed of light c_0, the polarization P, the acoustic damping term Γ' and the acoustic velocity v_{ac}. The polarization P and the driving term $\vec{f}$ of the

acoustic field can connected to electrostiction, giving equations (5) and (6), respectively.

$$P = \frac{1}{4\pi\rho_0} b\, E, \tag{5}$$

$$\vec{f} = -\gamma_{\mathrm{e}} \nabla \frac{\left\langle \vec{E} \cdot \vec{E} \right\rangle}{8\pi}. \tag{6}$$

With the density of the waveguide ρ_0 and the electrostrictive constant γ_{e}.

Applying a plain wave ansatz for both the optical and acoustic wave, gives a set of nonlinear partial differential equations. These cannot be solved analytically without applying several approximations. According to reference[9], one get a set of linear coupled partial differential equations by using the rotating frame and slowly-varying wave approximation, yielding the known coupled mode equations of the SBS process, which include two optical equations for the data wave a_{D} and the control wave a_{C} and one equation for the acoustic field b:

$$\begin{aligned}
\left(\frac{n_{\mathrm{eff}}}{c_0}\frac{\partial}{\partial t} + \frac{\partial}{\partial z}\right) a_{\mathrm{D}} &= -\mathrm{i}\frac{\gamma_{\mathrm{e}}\left(\omega_{\mathrm{C}} + \Omega\right)^2}{2\omega_{\mathrm{D}}\rho_0 c_0 n_{\mathrm{eff}}} a_{\mathrm{C}} b\, \mathrm{e}^{\mathrm{i}[(\omega_{\mathrm{C}}+\Omega)t-(q-k_{\mathrm{C}})z]} \\
\left(\frac{n_{\mathrm{eff}}}{c_0}\frac{\partial}{\partial t} - \frac{\partial}{\partial z}\right) a_{\mathrm{C}} &= -\mathrm{i}\frac{\gamma_{\mathrm{e}}\left(\omega_{\mathrm{D}} - \Omega\right)^2}{2\omega_{\mathrm{C}}\rho_0 c_0 n_{\mathrm{eff}}} a_{\mathrm{D}} b^*\, \mathrm{e}^{\mathrm{i}[(\omega_{\mathrm{D}}-\Omega)t-(k_{\mathrm{D}}-q)z]}, \\
\left(\frac{\partial}{\partial t} + v_{\mathrm{ac}}\frac{\partial}{\partial z} + \Gamma_{\mathrm{b}}\right) b &= -\mathrm{i}\frac{\gamma_{\mathrm{e}}\left(k_{\mathrm{D}} + k_{\mathrm{C}}\right)^2}{8\pi q v_{\mathrm{ac}}} a_{\mathrm{D}} a_{\mathrm{C}}^*\, \mathrm{e}^{\mathrm{i}[(\omega_{\mathrm{D}}-\omega_{\mathrm{C}})t-(k_{\mathrm{D}}+k_{\mathrm{C}})z]}.
\end{aligned} \tag{7}$$

With the effective refractive index of the waveguide n_{eff}, the speed of light c_0, the elecostrictive constant γ_{e}, the density of the waveguide ρ_0, the acoustic group velocity in the waveguide v_{ac}, the acoustic linewidth Γ_{b}, the optical wave vectors $k_{\mathrm{c,d}}$, the acoustic wave vector q and the frequency relation between the fields $\omega_{\mathrm{d}} = \omega_{\mathrm{c}} + \Omega + \Delta\omega$. Note that this set of equations capture also the acoustic phase introduced by an optical detuning from the Brillouin frequency: $\Delta\omega \neq 0$. The coupled mode equations can also be derived from the Hamiltonian given in equation (1) of the main text by applying the Heisenberg equation[14].

Table S2: Simulation parameters for the spectral-analysis of SBS.

Name	Symbol	Value	Reference
Elecostrictive const.	γ_{e}	$1\,\mathrm{m\,W^{-1}}$	[17]
Density	ρ_0	$2.2 \cdot 10^3\,\mathrm{g\,m^{-3}}$	[18]
Refractive index	n	1.44	[19]
Acoustic group velocity	v_{ac}	$6.3 \cdot 10^3\mathrm{m\,s^{-1}}$	[20]
Effective area	A_{eff}	$1.5 \cdot 10^{-12}\mathrm{m^{-2}}$	-
Acoustic lifetime	$\tau \propto \Gamma_{\mathrm{B}}^{-1}$	$8.8\,\mathrm{ns}$	-

Next, we numerically study the dynamics of the coupled mode equations (CME) (7) with the technique presented in Reference[15]. This method uses the characteristics of the data and control waves $\frac{n_{\mathrm{eff}}}{c_0} \pm z = 0$ and solves the CME along the $\frac{c_0}{n_{\mathrm{eff}}} t \pm z$-direction via an implicit Runge-Kutta scheme. As this approach only tackles the optical part of the CME, we also employ the Euler method[16] to solve the acoustic part of the CME. Therefore, we drop the space-derivative of the acoustic mode, assuming a stationary acoustic field. In order to run the simulation we feed the parameters shown in Table S1 into the numerical framework.

Supplementary Figure 8 depicts the simulation results of OREO in a highly nonlinear fiber. Although, we observe an overall agreement with the measurements results of the HNLF (see Fig. 5), the simulation show a linear behavior of the acoustic link for 2.5 ns (compare Fig. 5b and Fig. 8a).

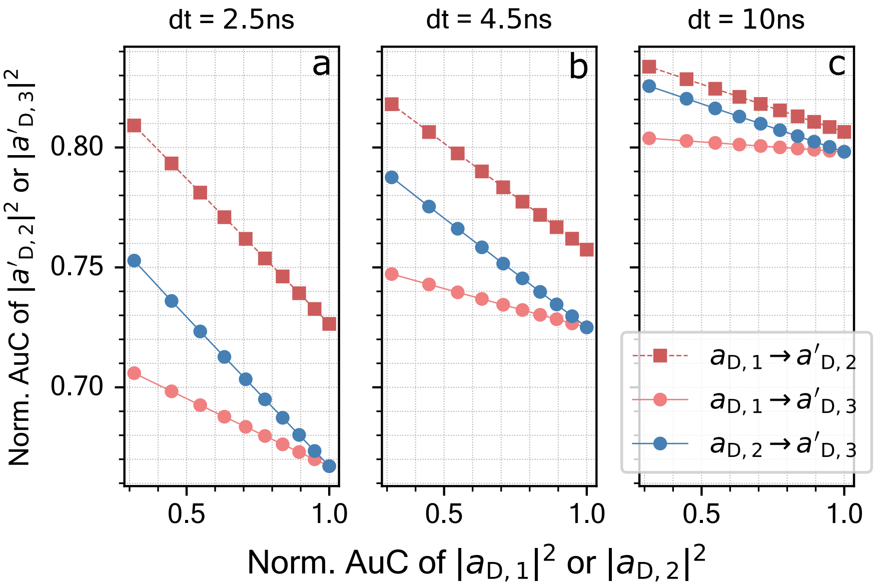

Supplementary Fig. 7: Numerical results of OREO's dynamic for a highly nonlinear fiber. One of the input data pulses $a_{\mathrm{D},1}$ or $a_{\mathrm{D},1}$ is swept in amplitude, while we study the impact on the output area under the curve (AuC) of the subsequent data pulses $a'_{\mathrm{D},2}$ and/or $a'_{\mathrm{D},3}$. The simulation results show agree almost completely with the measurements shown in Supplementary Figure 5.

S9 Numerical study of the *abc*-pattern prediction

In the following, we apply the simulation framework to study OREO's *abc*-pattern recognition performance in a highly nonlinear fiber (HNLF). In comparison to the *abc*-measurements, we operate OREO in-resonance ($\omega_{\mathrm{d}} = \omega_{\mathrm{c}} + \Omega + \Delta\omega$, $\Delta\omega = 0$) in order to evaluate OREO's fundamental response. We study the *abc*-pattern recognition task for different pulse lengths (pw), deadtimes (dt), acoustic lifetimes τ, and experimental precision. The first three variables are the input parameters of the simulation framework, whereas the latter one has to be considered in the post-processing. Therefore, we simulate OREO's dynamic for each of the 27 patterns considering a certain (pw, dt, τ)-setting. This yields 27 AuC-values for the Eval'-pulse, which serve as the mean value used to draw $n = 1000$

samples from a Gaussian distribution. The width of the Gaussian distribution is determined by the mean standard deviation acquired in the *abc*-pattern recognition experiment: $\overline{\sigma}_{\mathrm{exp}} = 0.13 \pm 0.01\,\mathrm{abr.u.}$. We numerically increase the precision of our experiment by decreasing $\overline{\sigma}_{\mathrm{exp}}$, e.g., doubling the precision corresponds to $\overline{\sigma}_{\mathrm{exp}}/2$. In total, we generate a dataset of size 27000, which we feed into a RandomForst (RFC) in the same way as in the experiment, which then returns a predictive accuracy.

Supplementary Figure 8 shows the results of the numerical study for different pulse widths, deadtimes, and experimental precision at each of the two acoustic lifetimes. The results suggest that the experimental precision has the largest impact on the RFC's accuracy. A five time increase in precision compared to the measurement results of the *ab*-measurement, which we discussed in the main text. In the most optimized case, the RFC achieves an accuracy of 92 % for the pulse width and deadtime used in the experiment. In addition, in the case of the standard acoustic lifetime of 8.8 ns as measured in the experiment (Fig. 8a), we observe that a shorter deadtime dt is beneficial for the performance of OREO. However, the influence of the investigated deadtime span decreases for the case of increased acoustic lifetime due to the slower acoustic decay. This explains why the different markers are less distributed in Supplementary Figure 8b. In addition, it suggests that a high acoustic lifetime could allow OREO to capture even longer patterns, i.e., more complex context carried by the optical domain. In this case, the precision of OREO can be pushed to 97 %

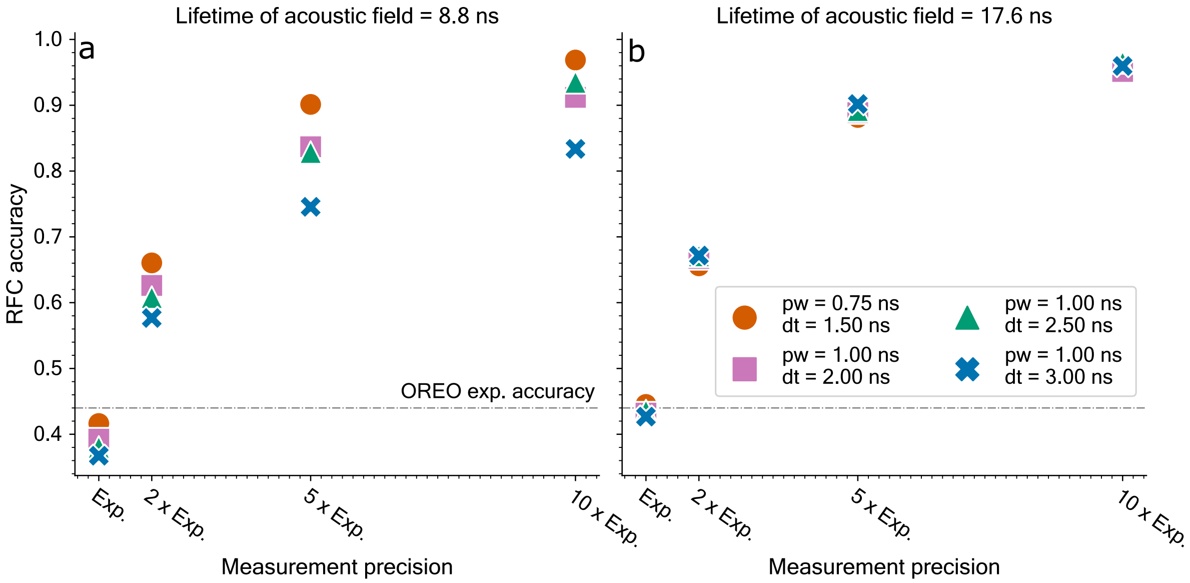

Supplementary Fig. 8: Numerical study of the *abc*-pattern prediction task. We generate a dataset by simulating OREO's behavior for the different 27-patterns, which gives an estimate for Eval'. Combined with the precision obtained in the experiment, we draw for each pattern 1000 times from a Gaussian distribution, where the estimate for Eval' represents its mean value. We perform this procedure for different pulse widths (pw), deadtimes (dt), and acoustic lifetimes. We simulate different experimental precision by reducing the value extracted from the experimental distributions, e.g., doubling the precision would correspond to $\overline{\sigma}_{\text{exp}}/2$. In the most optimized case OREO achieves together with the RFC an classification accuracy of 97 %.

S10 Comparing: OREO vs RNN

In the following, we compare the performance of OREO in the pattern recognition task with a digital recurrent neural network (RNN). OREO represents a single layer single neuron RNN, which serves us as a baseline for the digital RNN. We combine the digital RNN with a fully-connected neural network (FCN) in order to classify the input pattern. The FCN has one layer with 27 input and output neurons. The input neurons use ReLU as activation function and the output neurons use softmax activation. Analogous to our experiment, the digital RNN processes the sequential information and the FCN serves

as the classifier. While we keep the settings of the FCN constant, we train the RNNs different layers. As to compare the result with OREO, we use a single recurrent neuron per layer.

We generate a data set that represents the experimental inputs, using the following values for the different pulses, $a = 1$, $b = 0.5$, $c = 0.75$, and $Eval = 1$. We assume that the individual amplitudes follow a normal distribution with $\sigma = 0.019$ which represents an experimental value. For each pattern we sample $n = 5000$ input vectors. An exemplary input vector for the RNN is $[0.5, 0.5, 0.75, 1]$, where the last element 1 represents the evaluation pulse $Eval$ used for OREO. Supplementary Figure 9 depicts an exemplary data set without the evaluation pulse. We employ TensorFlow (v.2.11.0)[21] as underlying

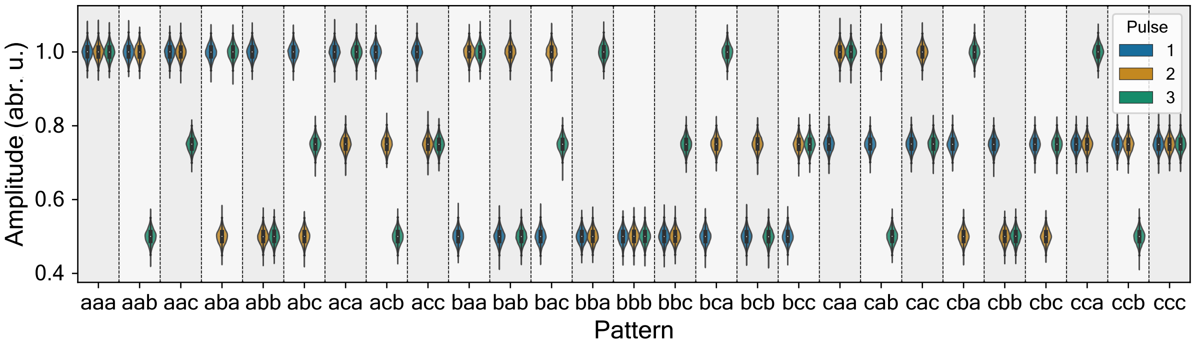

Supplementary Fig. 9: Violin plot of an exemplary data set used to train a digital RNN.

framework to train the digital neural network. We encode the ground truth patterns as one-hot vectors and use categorical crossentropy as the loss function. We utilize an Adam-optimizer with Early-Stopping option and a learning rate of 0.001. We train each model ten times with a different seed and a maximum number of epochs of 3500. We also performed studies with 10000, however found no improvement in the overall accuracy. While we are training the model, we use 15 % of the data set for validation. In addition, we track the best models with Tensorflow's ModelCheckpoint function. The best trained

model is tested with 30 % of the initial data set. We perform the splitting into the training, validation, and testing data set with the function train_test_split from the python-package Scikit-learn[22]. In order to compare the result with OREO, we average over the achieved test accuracy of the different models. The performance of the RNN and FCN improves for deeper network architectures, most probably originating with an improved sequential processing. Supplementary Figure 10 shows that the experimental

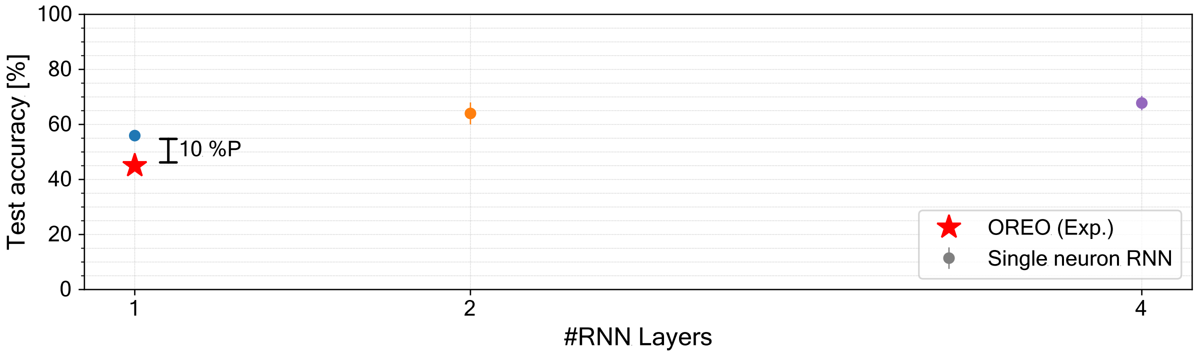

Supplementary Fig. 10: Comparing the performance of OREO with different digital RNNs.

realization of OREO is almost on par with the single-layer RNN and FCN combination. The single, double, and quadruple layer digital RNN achieves together with the FCN an test accuracy of 55.9 ± 0.2 %, 64.0 ± 4.0 %, and 67.8 ± 2.6 %, respectively. The larger uncertainties of the two and four layer case could be an indication for loss landscape with many local minima, which is also indicated by plotting the validation loss against the training epochs (see Supplementary Figure 11). In contrast to the RNN, we do not train OREO and use it as an extreme learner. Consequently, the accuracy of OREO and the RFC could be improved further by optimizing the amplitudes of the control pulses $a_{\mathrm{C,i}}$. This could be done in-situ with the stochastic gradient descent which have been discussed in[1].

In conclusion, this study showcase that OREO performs equivalent to a digital recur-

rent neuron and it can provide a classifier with the essential information in order to solve sequential tasks.

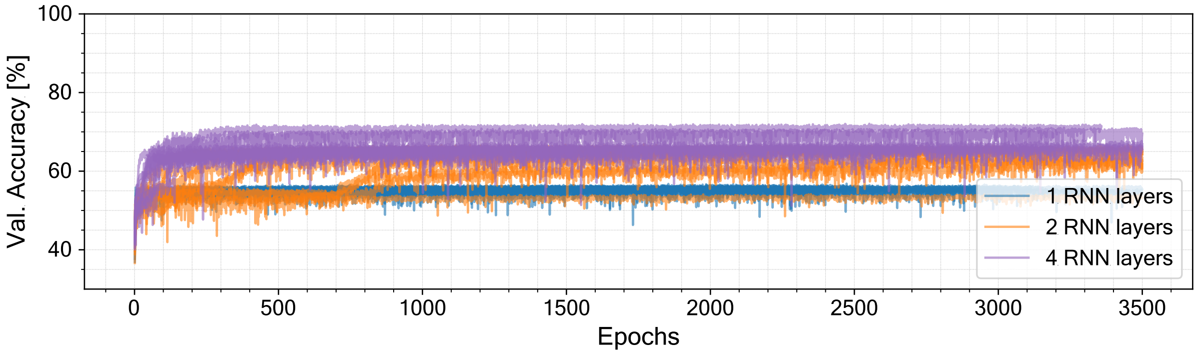

Supplementary Fig. 11: Illustration of the development of the validation accuracy over the epochs during the training.

References

[1] Bandyopadhyay, S. et al. Single chip photonic deep neural network with accelerated training (2022). ArXiv:2208.01623 [physics].

[2] Ashtiani, F., Geers, A. J. & Aflatouni, F. An on-chip photonic deep neural network for image classification. Nature 606, 501–506 (2022).

[3] Shen, Y. et al. Deep learning with coherent nanophotonic circuits. Nature Photonics 11, 441–446 (2017).

[4] Iwasaki, T. & Nemoto, T. A Homodyne Detection at 100 GHz with a Pyroelectric Detector. IEEE Transactions on Instrumentation and Measurement 29, 190–192 (1980).

[5] NewPort. Photoreceiver. URL https://www.newport.com/f/12-ghz-photoreceivers.

[6] Thorlabs. Thorlabs - RXM10AF Single Mode Ultrafast Receiver, 850 - 1650 nm, 40 kHz - 10 GHz, FC/PC. URL https://www.thorlabs.de.

[7] Zhu, Z., Gauthier, D. J. & Boyd, R. W. Stored Light in an Optical Fiber via Stimulated Brillouin Scattering. Science 318, 1748–1750 (2007).

[8] Merklein, M., Stiller, B., Vu, K., Madden, S. J. & Eggleton, B. J. A chip-integrated coherent photonic-phononic memory. Nature Communications 8, 574 (2017).

[9] Wolff, C., Smith, M. J. A., Stiller, B. & Poulton, C. G. Brillouin scattering—theory and experiment: tutorial. Journal of the Optical Society of America B 38, 1243 (2021).

[10] Boyd, R. W. Nonlinear optics (Academic Press, Amsterdam ; Boston, 2008), 3rd ed edn.

[11] Agrawal, G. P. Nonlinear fiber optics (Elsevier/Academic Press, Amsterdam, 2013), fifth edition edn.

[12] Eggleton, B. J., Steel, M. J. & Poulton, C. (eds.) Brillouin scattering. Part 1. No. volume 109 in Semiconductors and semimetals (Academic Press, an imprint of Elsevier, Cambridge, MA San Diego, CA Kidlington, Oxford London, 2022), first edition edn.

[13] Eggleton, B. J., Steel, M. J. & Poulton, C. (eds.) Brillouin scattering. Part 2. No. volume 110 in Semiconductors and semimetals (Academic Press, an imprint of Elsevier, Cambridge, MA San Diego, CA Kidlington, Oxford London, 2022), first edition edn.

[14] Zhang, J., Zhu, C., Wolff, C. & Stiller, B. Quantum coherent control in pulsed waveguide optomechanics. Physical Review Research 5, 013010 (2023).

[15] de Sterke, C. M., Jackson, K. R. & Robert, B. D. Nonlinear coupled-mode equations on a finite interval: a numerical procedure. Journal of the Optical Society of America B 8, 403 (1991).

[16] Butcher, J. C. Numerical methods for ordinary differential equations (Wiley, Chichester, 2005). OCLC: 835719460.

[17] Buckland, E. L. & Boyd, R. W. Electrostrictive contribution to the intensity-dependent refractive index of optical fibers. Optics Letters 21, 1117 (1996).

[18] Rumble, J. R. Density Ranges for Solid Materials. In CRC Handbook of Chemistry and Physics (CRC Press/Taylor & Francis, Boca Raton, FL USA, 2020), 101st edition edn.

[19] Malitson, I. H. Interspecimen Comparison of the Refractive Index of Fused Silica*,†. Journal of the Optical Society of America 55, 1205 (1965).

[20] Rumble, J. R. Speed of Sound in Various Media. In CRC Handbook of Chemistry and Physics (CRC Press/Taylor & Francis, Boca Raton, FL USA, 2020), 101st edition edn.

[21] Martín Abadi et al. TensorFlow: Large-Scale Machine Learning on Heterogeneous Systems (2015). URL https://www.tensorflow.org/.

[22] Pedregosa, F. et al. Scikit-learn: Machine Learning in Python. Journal of Machine Learning Research 12, 2825–2830 (2011). URL http://jmlr.org/papers/v12/pedregosa11a.html.
